# Zunahme von Bildungsungerechtigkeit durch die COVID-19-Pandemie beim Übergang in die Sekundarstufe II: Leistungsschwache und wenig motivierte Schüler*innen als besonders vulnerable Gruppe

**DOI:** 10.1007/s35834-022-00352-8

**Published:** 2022-08-12

**Authors:** Claudia Schreiner, Christian Kraler, Fred Berger, Wolfgang Hagleitner, Livia Jesacher-Rößler, Susanne Roßnagl

**Affiliations:** 1 Institut für LehrerInnenbildung und Schulforschung, Universität Innsbruck, Innsbruck, Österreich; 2 Institut für Erziehungswissenschaft, Universität Innsbruck, Innsbruck, Österreich

**Keywords:** Distanzunterricht, Selbstreguliertes Lernen, Bildungsgerechtigkeit, Personale, familiäre und schulische Ressourcen, Regressionsanalyse, Distance learning, Self-regulated learning, Educational equity, Personal, familial, and school-related resources, Regression analysis

## Abstract

Das Distanzlernen stellt an die Lernenden im Vergleich zum gewöhnlichen Schulalltag wesentlich höhere Anforderungen an deren Selbstregulationsfähigkeit. Zur erfolgreichen Bewältigung des Distanzlernens ist ein hohes Ausmaß an Selbstmanagement, Selbstmotivation und Selbstorganisation erforderlich. Hierbei können – entsprechend dem Ressourcenmodell der Lebensbewältigung (Fend et al. 2009) – familiäre, schulische und personale Ressourcen unterstützen. Basierend auf diesem theoretischen Zugang wurden 593 Schüler*innen, welche im Herbst 2021 in die weiterführenden Schulen und Ausbildungen der Sekundarstufe II gewechselt waren, zum Distanzlernen sowie dafür relevanten Ressourcen befragt. Diese hatten etwa einen Monat nach dem Wechsel eine viermonatige Distanzlernphase zu bewältigen. Im Rahmen der Datenanalyse wurden vier Regressionsmodelle berechnet, in die schrittweise die Merkmale einzelner im Beitrag beschriebener Ressourcenblöcke aufgenommen wurden. Die Analyse bestätigt insbesondere die große Bedeutung personaler Ressourcen (schulisches Leistungsniveau, Lernmotivation, allgemeine Selbstwirksamkeitsüberzeugung) für die Bewältigung der Anforderungen des Distanzunterrichts. Leistungsschwache und wenig motivierte Schüler*innen erweisen sich daher als besondere Risikogruppe im Kontext des Distanzunterrichts. Demnach benötigt diese vulnerable Gruppe sowohl in zukünftigen Phasen von Distanzunterricht als auch im Nachgang der Pandemie besondere Aufmerksamkeit und Unterstützung.

## Abstract (en)

When learning at home, students face higher demands in regards of self-regulated learning compared to learning at school. In order to cope with the demands of distance learning, high levels of self-management, self-motivation, and self-organization are required. This can be—according to the resource model of coping with developmental tasks (Fend et al. 2009)—supported by familial, school-related and personal resources. Based upon this theoretical approach, a survey was conducted with 593 students just after their transition to upper secondary schools in autumn 2021 on distance learning and relevant resources. They spent about one month in the new school before they had to cope with a 4-month long period of distance learning. Four regression models were estimated using step-wise inclusion of the different resources described in the paper. The analyses confirm above all the great importance of personal resources (academic achievement, learning motivation, general self-efficacy) for coping with the demands of distance learning. Low achievers and low motivated students prove to be particularly at risk in the context of distance learning. Therefore, this vulnerable group requires special attention and support both, in future periods of remote teaching as well as following the pandemic.

## Einleitung

Aus Sicht der Bildungsforschung liegt die Hypothese nahe, dass die Einschränkungen des normalen Präsenzschulbetriebs im Kontext der Covid-19-Pandemie eine verstärkende Wirkung auf Bildungsungerechtigkeit haben können (z. B. Grewenig et al. 2020; Huber und Helm 2020a; Jungkamp und Maaz 2021). Bis jetzt gibt es hierzu wenig belastbare Befunde (insbesondere für Österreich). Ziel der vorliegenden Studie war daher, die Bedeutung verschiedener Voraussetzungen von Jugendlichen für die Bewältigung des pandemiebedingten Distanzunterrichts zu beforschen, um zumindest indirekt Befunde für die Rolle von Distanzunterricht im Kontext von Bildungsgerechtigkeit^1^ zu generieren. Dabei steht vor allem das Konzept der Prozessgerechtigkeit (Fend 2019) im Mittelpunkt. Diese meint, „dass alle Kinder und Jugendlichen im Bildungswesen die gleichen Chancen haben sollten, optimale Angebote der Realisierung der eigenen Talente und Neigungen zu erfahren“ (Fend 2019, S. 110), auch in pandemiebedingten Phasen des Distanzunterrichts. Dem analytischen Vorgehen liegt die Hypothese zugrunde, dass (pandemiebedingter) Distanzunterricht ungerechtigkeitsverstärkend wirken kann, da unterschiedliche personale und familiäre Ressourcen im Distanzunterricht durch schulische Unterstützung nicht zur Gänze kompensiert werden können. Daher wird im vorliegenden Beitrag die Bedeutung personaler und familiärer sowie schulischer Ressourcen für die Bewältigung des pandemiebedingten Distanzunterrichts untersucht.

**Abb. 1:**
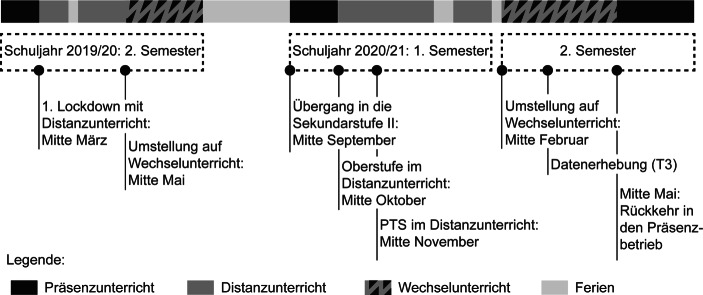
Unterrichtsformen zwischen dem zweiten Semester des Schuljahres 2019/20 und dem Ende des Schuljahres 2020/2021 (für die relevante Kohorte Mitte Schulstufe 8 bis Ende Schulstufe 9)

Die Analysen und Befunde der vorliegenden Untersuchung basieren auf einer Fragebogenstudie, die kurz nach der zweiten Phase mit Lockdowns und Distanzunterricht im Frühjahr 2021 durchgeführt wurde. Befragt wurden Jugendliche der neunten Schulstufe. Diese Kohorte wechselte während der Pandemie von der 8. in die 9. Schulstufe. Sie stand im Wintersemester 2020/21 vor einer doppelten Herausforderung: Erstens hatten die Jugendlichen die mit einem Schulwechsel verbundenen Herausforderungen zu meistern und zweitens wurde nach erst fünf Wochen Präsenzunterricht in der neuen Schule in den Distanzmodus gewechselt. Die neuen Lehrpersonen waren demnach noch wenig bekannt, genauso wie die mehrheitlich neuen Klassenkamerad*innen. Darüber hinaus dauerte die Phase des Distanzunterrichts für Oberstufenschüler*innen (in Tirol wie auch einigen anderen Bundesländern) mit etwa vier Monaten sehr lang. Für die Jugendlichen, die in Polytechnische Schulen (PTS) wechselten, galt dies in abgeschwächter Form, da hier in der Regel der Anteil bekannter Mitschüler*innen höher war und der Umstieg in den Distanzunterricht später erfolgte. Abb. 1 enthält eine Übersicht über die Phasen des Distanz‑, Präsenz- und Wechselunterrichts für die befragte Schüler*innen-Gruppe von Beginn der Pandemie im März 2020 bis zum Ende des Schuljahres 2020/21.

## Theoretischer Rahmen

Die theoretische Grundlage für die vorliegende Untersuchung bildet das Ressourcenmodell der Lebensbewältigung (Fend et al. 2009). In diesem werden sich wandelnde Umweltbedingungen und Ressourcenlagen mit der Bewältigung von Entwicklungsaufgaben im Lebenslauf in Verbindung gebracht. Die Bewältigung von Entwicklungsaufgaben im Lebensverlauf wird dabei als Ergebnis von Entscheidungsprozessen gesehen, die Jugendliche treffen müssen. Diese sind von den Möglichkeiten und Bedingungen des Umfelds abhängig und von den jeweiligen personalen und sozialen Ressourcen, die relevant dafür sind, welche Lebensmöglichkeiten aktiv genutzt werden (können) (Fend et al. 2009).

Im Kontext der vorliegenden Studie erwachsen zu bewältigende Aufgaben für die Jugendlichen aus der pandemiebedingten Umstellung des Unterrichts auf Distanzlernen. Die Pandemie stellt eine wesentliche Umweltbedingung dar. Dies steht in engem Zusammenhang mit der Lebensphase, in der sich die Jugendlichen befinden, dem Wechsel in die weiterführenden Schulen und Ausbildungen und den damit zusammenhängenden rechtlichen, organisatorischen und sozialen Rahmenbedingungen. In Anlehnung an das Ressourcenmodell (Fend et al. 2009) können unterschiedliche Arten von Ressourcen die Bewältigung des Distanzunterrichts und des Übergangs in weiterführende Schulen und Ausbildungen unterstützen. Eine wichtige Rolle spielen schulische und familiäre Ressourcen als spezifische soziale Ressourcen im Kontext des Distanzlernens sowie personale Ressourcen, auf welche die Jugendlichen bei der Bewältigung der an sie gestellten Herausforderungen zurückgreifen können.

Betrachtet man die Situation des Distanzlernens, so ergeben sich zentrale Herausforderungen aus dem Wegfallen der sozialen Faktoren in der schulischen Lernumgebung, den fehlenden oder eingeschränkten Möglichkeiten der Unterstützung durch die Lehrpersonen (Pelikan et al. 2021) sowie, daraus resultierend, ein schüler*innenseitig notwendiges hohes Ausmaß an Selbstorganisation, Selbststeuerung und Selbstmotivation im schulischen Lernen (Fischer et al. 2020). Selbstreguliertes Lernen meint dabei die Kompetenz der Lernenden, ihr Lernen autonom zu planen, durchzuführen und zu evaluieren (Wirth und Leutner 2008). Bei der Selbstregulation handelt es sich um einen dynamischen und zyklischen Prozess, der darin besteht, Aufgabenstellungen aktiv zu interpretieren, sich Ziele zu setzen, Pläne zu machen und Strategien zu finden, die den Erfolg sichern, indem die Zielerreichung kontinuierlich überwacht und Lernaktivitäten an die Zielerreichung angepasst werden (Schunk und Greene 2018). Die Bewältigung des Distanzunterrichts ist in wesentlichen Teilen davon bestimmt, wie gut es den Jugendlichen gelingt, mit den selbstregulatorischen Herausforderungen des Lernens zuhause zurecht zu kommen (Holzer et al. 2021; Letzel et al. 2020). Dafür stehen den Jugendlichen u. a. schulische, familiäre und personale Ressourcen zur Verfügung. Vor dem Hintergrund der Situation des pandemiebedingten Distanzlernens können diese drei Arten von Ressourcen wie folgt operationalisiert werden.

Erstens gewinnt *das familiäre Umfeld* unter den Bedingungen des Distanzlernens an Bedeutung. Das bezieht sich sowohl auf die sozioökonomische Lage der Familie (in diesem Beitrag operationalisiert über den höchsten Bildungsabschluss der Eltern) als auch auf die konkreten Bedingungen, die Jugendliche für das Lernen zuhause vorfinden (Helm et al. 2021), sowie die Beziehungsqualität zwischen Eltern und Kind im Sinn einer emotionalen Stütze (Responsivität im Erziehungsverhalten der Eltern).

Zweitens sind *schulische Ressourcen* ein wichtiges Element für die Gestaltung und Unterstützung des Distanzunterrichts. Ihnen kommt insbesondere auch eine ausgleichende Funktion vor dem Hintergrund unterschiedlicher familiärer Ressourcen zu. Im vorliegenden Beitrag werden schulische Ressourcen über die Erreichbarkeit der Lehrpersonen (aus der Perspektive der Schüler*innen) als konkrete Unterstützung im Kontext des Distanzunterrichts (Huber et al. 2020), das Ausmaß an Unterstützungsverhalten durch die Lehrpersonen allgemein sowie durch einen allgemeinen Indikator dafür, wie gut die Jugendlichen bereits in der neuen Schule angekommen sind und Vertrauen zu ihren neuen Lehrer*innen aufbauen konnten, erfasst. Letzteres wird negativ über das Ausmaß an Entfremdung von Lehrpersonen (Hascher und Hadjar 2018) operationalisiert. Diese bildet ein Kernelement im Modell der Schulentfremdung, welches den Prozess einer wachsenden Distanzierung von spezifischen Objekten des schulischen Umfelds beschreibt (Morinaj et al. 2020). Entfremdung von Lehrpersonen äußert sich in der Geringschätzung einer positiven Beziehung zu Lehrpersonen sowie mangelndem Vertrauen in und fehlender wahrgenommener Wertschätzung durch die Lehrpersonen (Hascher und Hadjar 2018).

Drittens werden als *personale Ressourcen* kognitive Ressourcen (schulische Leistungsfähigkeit) und anforderungsbezogene Haltungen spezifiziert. Bezogen auf die Haltung wird sowohl die schulische Lernmotivation als auch die allgemeine Selbstwirksamkeitsüberzeugung berücksichtigt. Beide stehen eng mit dem selbstregulierten Lernen in Verbindung (Perry et al. 2018; Zimmerman und Schunk 2011). In Anlehnung an frühere Forschungsarbeiten wird davon ausgegangen, dass die Bewältigung des Distanzlernens insbesondere mit dem Leistungsniveau und der Lernmotivation der Schüler*innen in Verbindung steht, da leistungsschwächere und wenig motivierte Schüler*innen eher hochstrukturierte Lernumgebungen benötigen und motivierte Schüler*innen von geöffneten Unterrichtsformaten profitieren (Fischer et al. 2020; Perry et al. 2018).

Zur Frage von möglichen ungerechtigkeitsverstärkenden Effekten der pandemiebedingten Unterbrechungen des Präsenzschulbetriebs liegen zwischenzeitlich vereinzelt Studien aus dem europäischen Ausland, vorrangig bezogen auf die Primarstufe, vor. Während Depping et al. (2021) für Hamburg keine relevanten Lernverluste durch den ersten Lockdown im Frühjahr 2020 dokumentieren, zeigen Untersuchungen in anderen Ländern Lernverluste durch die Schulschließungen, die insbesondere für benachteiligte bzw. leistungsschwache Schüler*innen umfangreich ausfielen (Blainey et al. 2020 für England; Engzell et al. 2021 für die Niederlande; Maldonado und De Witte 2021 für Belgien; jeweils für die Primarstufe). Tomasik et al. (2021) dokumentieren für die Schweiz eine steigende Leistungsheterogenität für Volksschulen, nicht jedoch in der Sekundarstufe, ohne allerdings einen spezifischen Bezug zu Ungleichheitsdimensionen herzustellen.

Der vorliegende Beitrag ergänzt den aktuellen, primär auf das Lernen in Primarstufen fokussierten Forschungsstand, indem die Relevanz der verschiedenen Ressourcenarten gemeinsam modelliert und eine ältere Schüler*innen-Gruppe in den Blick genommen wird. In bestehenden Studien werden potenzielle Ressourcen für die Bewältigung entweder deskriptiv beschrieben oder getrennt voneinander untersucht (Helm et al. 2021). Die diesem Beitrag zugrundeliegenden Forschungsfragen lauten darauf aufbauend:

1. Wie wirken sich familiäre, schulische und personale Ressourcen auf die Bewältigung des Distanzunterrichts aus?
2. Wie unterscheiden sich familiäre, schulische und personale Ressourcen in ihrer Erklärungskraft für die Bewältigung des Distanzunterrichts?

Vor dem Hintergrund potenziell ungerechtigkeitsverstärkender Wirkung des Distanzunterrichts ist dabei insbesondere die Bedeutung personaler und familiärer Ressourcen für die Bewältigung des Distanzunterrichts von Interesse.

## Methoden

### Forschungskontext, Erhebungsmethode und Stichprobe

Die im Beitrag präsentierten Ergebnisse basieren auf einer Längsschnittstudie, die in einer ländlichen Region Tirols die schulische Entwicklung und den Übergang in weiterführende Ausbildungen einer Kohorte von Jugendlichen von der 7. bis zur 9. Schulstufe untersucht. Diese Studie ist Teil eines größeren Schulentwicklungsprojekts (Modellregion Bildung Zillertal; Kraler und Rößler 2016). Im Zentrum des Interesses stehen die Übergänge im Bildungssystem und Bedingungen sowie Ressourcen, die die Bewältigung von Übergangen begünstigen. Sie wurde im Mai 2019 (T1) mit den Schüler*innen einer vollständigen Kohorte der Region begonnen und während der Zeit rund um den Übergang von der Sekundarstufe I in die weiterführenden Schul- und Bildungsangebote der Sekundarstufe II jährlich weitergeführt (T2: Juli 2020; T3: April 2021). Der dritte Befragungszeitraum (T3), der die Grundlage für den vorliegenden Beitrag bildet, fiel in den Zeitraum des pandemiebedingten Wechselunterrichts im Frühjahr 2021. Lernen unter Bedingungen der Pandemie wurde aufgrund der Umstände ab T2 als Thema in die Befragungen integriert und konzeptuell mit dem der Studie zugrunde gelegten Ressourcenmodell verknüpft. Alle Erhebungen wurden mittels Paper-Pencil-Fragebögen durchgeführt.

Die Schulstichprobe für T3 umfasst jene 12 Schulen, in die Schüler*innen der untersuchten Region in der Regel nach der Mittelschule wechseln (3 Polytechnische Schulen (PTS), 3 Berufsbildende Mittlere Schulen (BMS), 3 Allgemeinbildende Höhere Schulen (AHS), 3 Berufsbildende Höhere Schulen (BHS); Rücklauf auf Schulebene: 100 %). Innerhalb dieser Schulen wurden jeweils Vollerhebungen in der 9. Schulstufe durchgeführt. T3 umfasst insgesamt 593 Schüler*innen aus 30 Klassen (Rücklauf auf Individualebene: 83,4 %). In Tab. 1 ist die Zusammensetzung der Stichprobe dargestellt. Hierbei ist auf einen etwas erhöhten Anteil an Mädchen hinzuweisen sowie auf den relativ niedrigen Anteil an Schüler*innen mit anderen Erstsprachen als Deutsch, wobei letzteres mit den regionalen Gegebenheiten der untersuchten Region zusammenhängt (Berger et al. 2021).

**Tab. 1:** Demografische Merkmale der Stichprobe

*Geschlecht, n (%)*		
Weiblich	320	(54,0 %)
Männlich	262	(44,2 %)
Keine Angabe	11	(1,9 %)
*Alter*		
M (SD)	16,0	(0,82)
Keine Angabe	7	(1,2 %)
*Erstsprache, n (%)*		
Deutsch	529	(89,2 %)
Ausschließlich andere Sprache(n)	59	(9,9 %)
Keine Angabe	5	(0,8 %)
*Aktuell besuchte Schulart, n (%)*		
Polytechnische Schule (PTS)	112	(18,9 %)
Berufsbildende Mittlere Schule (BMS)	153	(25,8 %)
Allgemein- oder Berufsbildende Höhere Schule (ABHS)	328	(55,3 %)
*Bildung der Eltern (höchster Abschluss der beiden Elternteile), n (%)*		
Max. Pflichtschule	30	(5,1 %)
Berufsausbildung	343	(57,8 %)
Schule mit Matura	108	(77,9 %)
Universitäre Ausbildung o. ä.	89	(15,6 %)
Keine Angabe	23	(3,9 %)

### Instrumente

Der Fragebogen für die 9. Schulstufe umfasst demografische Merkmale der Jugendlichen, Informationen zu personalen, schulischen sowie familiären Ressourcen, zum Übergang in die Sekundarstufe II und zur Bewältigung des Distanzunterrichts. Dabei wurde der Fragebogen im Kontext der Längsschnittstudie konzipiert, wobei auf Konzepte und Skalen vor allem aus der LifE-Studie (Fend et al. 2012), der NMS-Evaluation (Eder et al. 2015), COACTIV (Baumert et al. 2008), Forschung zur Schulentfremdung (Morinaj et al. 2020) und des ThüBOM-Projekts (Lipowski et al. 2015) zurückgegriffen wurde.

Für die Analysen des vorliegenden Beitrags werden folgende Kontrollvariablen verwendet: das Geschlecht (dummy-kodiert, weiblich = 1), die Erstsprache (dummy-kodiert, Deutsch und ggf. weitere Erstsprachen = 1) sowie die aktuell besuchte Schulart (dummy-kodiert, Referenzkategorie PTS: Polytechnische Schule; BMS: Berufsbildende Mittlere Schule; ABHS: Allgemeinbildende oder Berufsbildende Höhere Schule). Der *höchste Bildungsabschluss der Eltern* wird – dem Ressourcenmodell zur Lebensbewältigung (Fend et al. 2009, S. 17) entsprechend – als familiäre Ressource interpretiert. Verwendet wird in Anlehnung an den nationalen Bildungsbericht (Neubacher et al. 2019) jeweils der höhere Formalabschluss der beiden Elternteile in vier Kategorien: max. Pflichtschulabschluss, Berufsausbildung (Lehre, Berufsbildende Mittlere Schule), Schule mit Matura (Allgemeinbildende oder Berufsbildende Höhere Schule), Universitätsabschluss oder Vergleichbares.

Die abhängige Variable der Analysen bildet die Skala „Bewältigung des Distanzunterrichts“. Mit dieser wird erhoben, wie die Schüler*innen ihre Bewältigung der durch den Distanzunterricht bedingten selbstregulatorischen Anforderungen wahrgenommen haben. Diese Skala wurde für die Erhebung zu T2 im Frühjahr 2020 von den Autor*innen entwickelt (Berger et al. 2021) und im Zuge der Erhebung zu T3 im Frühjahr 2021 erweitert. Sie besteht nun aus 6 Items mit 4‑ bzw. 5‑stufigen Likert-Skalen. Alle Items wurden auf den Wertebereich 0 bis 3 transformiert und der Skalenwert als Mittelwert der Items berechnet. Negativ formulierte Items wurden umgepolt, sodass hohe Werte für ein hohes Bewältigungsempfinden stehen. Zentrale Kennwerte für die Skala Bewältigung des Distanzunterrichts sowie alle weiteren in den Analysen verwendeten Konstrukte sind in Tab. 2 dokumentiert.

**Tab. 2:** Verwendete Skalen und Konstrukte

Skala	Beispielitem	Anzahl Items	M (SD)	Missing in %	Cron-bachs α
*Bewältigung des Distanzunterrichts*	„Es war für mich schwierig meinen Lernalltag zu strukturieren.“ (rec.) (0 – nie bis 3 – oft)	6	1,68 (0,70)	1,3	0,817
*Ausstattung für den Distanzunterricht*	Technische Ausstattung sowie Lernumgebung zuhause: „Bei uns zu Hause gab es einen Ort, an dem ich ungestört meine Aufgaben erledigen konnte.“ (1 – stimmt nicht bis 4 – stimmt genau)	7	3,51 (0,53)	2,2	0,799
*Responsivität – Verständnis und Unterstützung im Erziehungsverhalten der Eltern *(Berger und Fend 2005)	„Mein Vater/meine Mutter hört immer aufmerksam zu, wenn ich etwas erzähle.“ (1 – stimmt gar nicht bis 5 – stimmt völlig)	8	4,09 (0,83)	1,0	0,899
*Erreichbarkeit der Lehrpersonen*	„Ich konnte meine Lehrerinnen und Lehrer gut erreichen.“ (1 – nie bis 4 – oft)	1	3,25 (0,78)	2,5	–
*Teacher Support *(adaptiert von OECD 2004)	„Auf wie viele Lehrer/innen treffen die Aussagen zu?“ z. B. „Unsere Lehrer/innen erklären etwas so lang, bis wir es verstehen.“ (1 – auf keine oder gar keine bis 4 – auf alle oder fast alle)	5	2,7 (0,69)	0,8	0,811
Schulentfremdung – Entfremdung von Lehrer*innen (Morinaj et al. 2020)	„Ich fühle mich nicht ernst genommen von meinen Lehrer/innen.“ (1 – stimmt nicht bis 4 – stimmt genau)	7	1,8 (0,62)	1,0	0,839
*Schulisches Leistungsniveau*	Mittelwert der Noten aus dem letzten Semesterzeugnis (9. Schulstufe) in Deutsch, Mathematik und Englisch so transformiert, dass hohe Werte für ein hohes Leistungsniveau stehen	3	3,30 (0,96)	1,0	–
*Schulische Lernmotivation *(Fend und Prester 1986)	„Wie sehr strengst du dich für die Schule an?“ (1 – gar nicht bis 4 – sehr)	3	2,75 (0,69)	0,8	0,802
*Allgemeine Selbstwirksamkeitsüberzeugung *(Jerusalem und Schwarzer 2013)	„Für jedes Problem kann ich eine Lösung finden.“ (1 – stimmt nicht bis 4 – stimmt genau)	7	2,67 (0,57)	0,5	0,842

### Datenanalyse

Ziel der Analyse ist auf Basis der Forschungsfragen die Schätzung des Einflusses familiärer, schulischer und personaler Ressourcen auf die wahrgenommene Bewältigung des Distanzunterrichts. Dazu wurden Regressionsmodelle berechnet, in die schrittweise die Merkmale zu den einzelnen Ressourcenblöcken aufgenommen wurden. Alle Modelle wurden auf Basis der gleichen Stichprobe (*N* = 529) berechnet, für welche bei allen verwendeten Merkmalen gültige Werte vorlagen. Mit den familiären und schulischen Ressourcen werden die Kontextbedingungen für die Entwicklung von personalen Ressourcen erhoben. Sie werden deshalb als erste Blöcke ins Modell eingeführt. Mit der Aufnahme der personalen Ressourcen im letzten Block wird darüber hinaus ermöglicht, die explanative Kraft der personalen Ressourcen bei Konstanthalten aller Kontextbedingungen (inkl. der familiären und schulischen Ressourcen) zu schätzen. Um das Ausmaß der explanativen Kraft der personalen Ressourcen mit dem der anderen Ressourcengruppen vergleichen zu können (vgl. Forschungsfrage 2), wurden die Regressionsanalysen darüber hinaus schrittweise mit unterschiedlicher Reihenfolge der Ressourcenblöcke erneut durchgeführt, um so die unique Varianzaufklärung der verschiedenen Blöcke schätzen zu können.

Die Regressionsmodelle (vgl. Tab. 3) wurden auf Individualebene geschätzt, da es sich bei den potenziellen Einflussfaktoren primär um individuelle oder von den Schüler*innen individuell wahrgenommene Merkmale handelt. Aufgrund der kleinen Anzahl an Fällen auf Schulebene war eine Mehrebenenanalyse nicht möglich (Snijders 2015). Um Vergleiche zwischen den Koeffizienten der einzelnen Variablen zu erlauben, werden standardisierte β‑Koeffizienten berichtet. Alle Analysen wurden im Programm Mplus (Muthen und Muthen 2017) mittels Bootstrap-Methode (Bootstrap N = 10.000) berechnet, um bias-korrigierte Standardfehler (bei teilweiser Verletzung der Normalverteilungsannahme) zu erhalten.

**Tab. 3:** Ergebnisse der Regressionsanalysen

	Modell 1		Modell 2		Modell 3		Modell 4	
	β	*(SE)*	β	*(SE)*	β	*(SE)*	β	*(SE)*
**Kontrollvariablen:**								
Besuchte Schulart – BMS^a^	**−0,193**	*(0,057)* **	**−0,213**	*(0,052)* ***	**−0,175**	*(0,050)* **	**−0,244**	*(0,049)* ***
Besuchte Schulart – ABHS^a^	−0,045	*(0,056)*	**−0,110**	*(0,050)* *	**−0,118**	*(0,050)* *	**−0,170**	*(0,048)* ***
Geschlecht (weiblich)	0,046	*(0,044)*	0,048	*(0,041)*	−0,011	*(0,041)*	**−0,077**	*(0,038)* *
Erstsprache (Deutsch)	**0,083**	*(0,038)* *	0,013	*(0,038)*	0,022	*(0,036)*	0,012	*(0,032)*
**Familiäre Ressourcen:**								
Bildung der Eltern – Beruf^b^	–	–	−0,007	*(0,090)*	−0,066	*(0,080)*	−0,126	*(0,074)*
Bildung der Eltern – Matura^b^	–	–	−0,057	*(0,077)*	−0,111	*(0,069)*	**−0,160**	*(0,064)* *
Bildung der Eltern – Universität^b^	–	–	−0,022	*(0,076)*	−0,047	*(0,068)*	−0,122	*(0,063)*
Ausstattung zuhause für den Distanzunterricht	–	–	**0,344**	*(0,038)* ***	**0,236**	*(0,039)* ***	**0,168**	*(0,039)* ***
Responsivität im Erziehungsverhalten der Eltern	–	–	**0,100**	*(0,042)* *	−0,016	*(0,046)*	−0,039	*(0,044)*
**Schulische Ressourcen:**								
Erreichbarkeit der Lehrpersonen im Distanzunterricht	–	–	–	–	**0,174**	*(0,042)* ***	**0,139**	*(0,038)* ***
Teacher Support	–	–	–	–	0,041	*(0,047)*	−0,001	*(0,043)*
Schulentfremdung – Entfremdung von Lehrpersonen	–	–	–	–	**−0,232**	*(0,045)* ***	**−0,159**	*(0,042)* ***
**Personale Ressourcen:**								
Schulisches Leistungsniveau	–	–	–	–	–	–	**0,226**	*(0,043)* ***
Schulische Lernmotivation	–	–	–	–	–	–	**0,243**	*(0,046)* ***
Selbstwirksamkeitsüberzeugung	–	–	–	–	–	–	**0,115**	*(0,041)* **
F‑Wert	2,013		6,087		8,429		12,972	
R^2^	0,031*		0,173***		0,263***		0,399***	

Abhängige Variable: Bewältigung des Distanzunterrichts

N (Modelle 1–4) = 529

^a^ Referenzgruppe: PTS

^b^ Referenzgruppe: max. Pflichtschule

* *p* < 0,05; ** *p* < 0,01; *** *p* < 0,001

## Ergebnisse

Regressionsmodell 1 zeigt in einem ersten Schritt den Zusammenhang der Kontrollvariablen mit der abhängigen Variable „Bewältigung des Distanzunterrichts“ (vgl. Tab. 3). Schüler*innen, die in eine BMS gewechselt sind, hatten demnach mehr Probleme bei der Bewältigung des Distanzunterrichts als die der Referenzgruppe in PTS (β = −0,193; *p* =0,001). Schüler*innen mit Deutsch als Erstsprache fiel die Bewältigung des Distanzunterrichts etwas leichter als jenen mit anderen Erstsprachen (β = 0,083; *p* =0,029). Zwischen Mädchen und Jungen sowie Schüler*innen in höheren Schulen und solchen in der Referenzgruppe PTS sind keine Unterschiede zu beobachten. Modell 1 ist statistisch signifikant (F = 2,013; *p* =0,044), die erklärte Varianz mit 3,1 % jedoch sehr gering.

In Modell 2 wurden familiäre Ressourcen hinzugenommen. Die Varianzaufklärung steigt deutlich auf 17,3 % (F = 6,087; *p* < 0,001). Während für das Bildungsniveau der Eltern keine Effekte nachgewiesen werden können, erweisen sich sowohl die zuhause vorhandene Ausstattung für den Distanzunterricht (β = 0,344; *p* < 0,001) als auch ein von Verständnis und Unterstützung geprägtes Erziehungsverhalten der Eltern (β = 0,100; *p* =0,019) als relevante Einflussfaktoren. Bei Berücksichtigung der familiären Ressourcen wird zusätzlich der Unterschied in der wahrgenommenen Bewältigung zwischen Schüler*innen in höheren Schulen und jenen in der Referenzgruppe PTS signifikant und zwar in Richtung besseres Bewältigungsempfinden in der PTS (β = −0,110; *p* =0,028).

Modell 3 berücksichtigt zusätzlich schulische Ressourcen für die Bewältigung des Distanzunterrichts. Dies führt zu einer weiteren Zunahme der erklärten Varianz um knapp 10 Prozentpunkte auf insgesamt 26,3 % (F = 8,429; *p* < 0,001). Als signifikante Faktoren zeigen sich die Erreichbarkeit der Lehrpersonen während des Distanzunterrichts (β = 0,174; *p* < 0,001) sowie die Entfremdung von Lehrpersonen (als Maß einer (noch) nicht etablierten Beziehung zu den neuen Lehrpersonen und unzureichender Einbettung in den schulischen Kontext; β = −0,232; *p* < 0,001). Das Erziehungsverhalten der Eltern verliert bei Einbeziehung der schulischen Ressourcen an Erklärungskraft und erweist sich in Modell 3 nicht mehr als signifikant.

In Modell 4 werden schließlich darüber hinaus personale Ressourcen einbezogen. Das führt zu einer Steigerung der erklärten Varianz auf 39,9 % (F = 12.972; *p* < 0,001). Somit erweisen sich die personalen Ressourcen als sehr erklärungsstark. Alle drei Merkmale zu personalen Ressourcen weisen einen signifikanten Einfluss auf das Bewältigungsempfinden auf: das schulische Leistungsniveau (β = 0,226; *p* < 0,001), die schulische Lernmotivation (β = 0,243; *p* < 0,001) sowie die allgemeine Selbstwirksamkeitsüberzeugung (β = 0,115; *p* =0,005). Bei konstant gehaltenen personalen Ressourcen ist ein kleiner Unterschied im Bewältigungsempfinden zwischen Mädchen und Jungen (β = −0,077; *p* =0,040) zugunsten letzterer zu beobachten. Der Unterschied zwischen Jugendlichen, deren Eltern Matura abgeschlossen haben, und solchen mit formal sehr niedrig qualifizierten Eltern wird signifikant (β = −0,160; *p* =0,012) und die schulischen Ressourcen verlieren etwas an Erklärungskraft (Erreichbarkeit der Lehrpersonen: β = 0,139 und Schulentfremdung: β = −0,159), bleiben jedoch signifikant (jeweils *p* < 0,001).

**Abb. 2:**
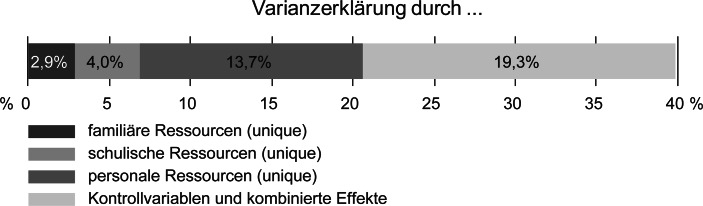
Anteile erklärter Varianz nach Ressourcenblöcken des Regressionsmodells

Die Varianzaufklärung, die unique auf die personalen Ressourcen zurückzuführen ist, beträgt 13,7 %. Eine Variation der Reihenfolge der verschiedenen Ressourcenblöcke ergibt für die im Modell berücksichtigten familiären Ressourcen 2,3 % und für die schulischen 4,0 % Varianzaufklärung. Die restlichen knapp 20 % entfallen auf die Kontrollvariablen sowie kombinierte Effekte der verschiedenen Ressourcen (vgl. Abb. 2).

## Diskussion

Die Analysen verdeutlichen bemerkenswerte Unterschiede in der Wahrnehmung der Bewältigung des Distanzunterrichts zwischen den Schularten. So erleben sich die Schüler*innen in Polytechnischen Schulen erfolgreicher im Distanzunterricht als Schüler*innen in mittleren und höheren Schulen. Dies scheint im Widerspruch zu stehen mit dem allgemeinen Befund, dass leistungsstärkere Schüler*innen mit dem Distanzunterricht im Schnitt besser zurechtkommen, wechseln doch in der Regel Jugendliche mit vergleichsweise weniger hohem individuellem Leistungsniveau in die Polytechnischen Schulen. Eine mögliche Erklärung könnte darin bestehen, dass nicht die absolute Bewältigung gemessen wird, sondern die persönliche Wahrnehmung der Bewältigung. Dabei ist vermutlich relevant, dass durch den Schulwechsel der Referenzrahmen, den die Schüler*innen als Vergleichsbasis zur Einschätzung ihrer eigenen Bewältigung heranziehen, erweitert worden ist. Darüber hinaus fließt das Anforderungsniveau der jeweiligen Schule (auch im Vergleich zum bekannten Anforderungsniveau der Mittelschule) ein, das in der Regel in mittleren und höheren Schulen höher ist als in Polytechnischen Schulen. Ein weiterer Erklärungsansatz könnte im Bereich der metakognitiven Kompetenz liegen, die bei Schüler*innen höherer Schulen im Schnitt stärker ausgeprägt ist, was zu kritischeren Einschätzungen der eigenen Leistungen führt. Allerdings wurden im Rahmen der vorliegenden Studie keine Informationen über die Gestaltung der Lernprozesse im Distanzunterricht erhoben, was eine Limitation der Studie ist. Es ist davon auszugehen, dass die konkrete Ausgestaltung an den verschiedenen Schulstandorten deutlich variierte (Steiner et al. 2021). Ob dies relevant für die beobachteten Unterschiede in der Bewältigung des Distanzunterrichts durch Schüler*innen verschiedener Schularten ist, kann auf Basis der vorhandenen Datenbasis nicht beurteilt werden.

Vor dem Hintergrund der deutlich positiveren Wahrnehmung der Bewältigung des Distanzunterrichts durch Mädchen in der gleichen Kohorte am Ende der 8. Schulstufe (Berger et al. 2021) überrascht die etwas niedrigere Einschätzung der eigenen Bewältigung der Mädchen in der 9. Schulstufe aus den vorliegenden Analysen. Untersucht wird im vollständigen Regressionsmodell (Modell 4) die Bewältigung des Distanzunterrichts jeweils in der gleichen Schulart sowie insbesondere bei jeweils gleichen personalen Ressourcen. Zudem liegt den Analysen die persönlich wahrgenommene Bewältigung, nicht ein objektives Maß der Bewältigung des Distanzunterrichts zugrunde. Aus der Forschung ist bekannt, dass Mädchen im Schnitt dazu neigen, ihre Leistungen zu unterschätzen, während Jungen im Schnitt zur Überschätzung neigen (vgl. dazu etwa Schreiner et al. 2019). Zu unterschiedlichen Befunden bezüglich des Geschlechts kommen auch andere Studien, etwa Korlat et al. (2021), die keine Unterschiede zwischen Mädchen und Jungen beobachten, während Grewenig et al. (2020) indirekt über die höhere Lernmotivation und Beschäftigungszeit mit schulischen Aufgaben von Mädchen auf deren bessere Bewältigung schließen.

Bezüglich der Wirkung unterschiedlicher Ressourcen auf die Bewältigung des Distanzunterrichts (Forschungsfrage 1) zeigen sich relevante Zusammenhänge bei allen Ressourcenarten.

Bei den familiären Ressourcen spielt das Bildungsniveau der Eltern eine geringe Rolle, die im familiären Haushalt für den Distanzunterricht verfügbare Ausstattung erweist sich demgegenüber jedoch von großer Bedeutung (β = 0,168; *p* < 0,001). Dies deckt sich mit Ergebnissen anderer Studien, die die Bedeutung eines geeigneten Platzes zum Lernen (Schwerzmann und Frenzel 2020) sowie jene der technischen Ausstattung (Huber und Helm 2020b) herausstreichen. Die Beziehungsqualität mit den Eltern (wahrgenommene Responsivität) erweist sich nur in Modell 2 (ohne Berücksichtigung schulischer oder personaler Ressourcen) als signifikant.

Bei den schulischen Ressourcen zeigen sich – auch bei Berücksichtigung familiärer und personaler Ressourcen in Modell 4 – die von den Schüler*innen wahrgenommene Erreichbarkeit der Lehrpersonen während der Distanzunterrichtsphase (β = 0,139; *p* < 0,001) sowie die Beziehungsqualität zu den Lehrpersonen (β = −0,159; *p* < 0,001 für die Entfremdung von Lehrpersonen) als relevante Prädiktoren für die Bewältigung des Distanzunterrichts.

Als besonders gute Prädiktoren erweisen sich die personalen Ressourcen. Schüler*innen mit guten Noten (β = 0,226; *p* < 0,001) und hoher Lernmotivation (β = 0,243; *p* < 0,001) bewältigen die Anforderungen des Distanzunterrichts besser. Damit sind leistungsstarke und hoch motivierte Schüler*innen erwartungskonform erfolgreicher im Distanzunterricht, womit Befunde aus anderen Ländern zur Primarstufe (siehe Abschn. 2) auch für ältere Schüler*innen gestützt werden. Darüber hinaus wirkt sich eine hohe allgemeine Selbstwirksamkeitsüberzeugung (β = 0,115; *p* = 0,005) ebenfalls positiv auf die Bewältigung des Distanzunterrichts aus, was im Einklang mit Befunden zum selbstregulierten Lernen (z. B. Perry et al. 2018) steht.

Im Vergleich der Erklärungskraft zwischen den verschiedenen Ressourcenblöcken (Forschungsfrage 2) erweisen sich die personalen Ressourcen als für sich genommen erklärungsstärkster Block. Bei Konstanthalten aller anderen berücksichtigten Ressourcen kommen ihnen 13,7 % Varianzaufklärung zu. Damit ist die unique den im Modell berücksichtigten personalen Ressourcen zurechenbare Varianzaufklärung wesentlich höher als jene der anderen Ressourcenblöcke mit 2,3 % bei den familiären und 4,0 % bei den schulischen Ressourcen.

## Limitationen

Die vorliegende Studie basiert auf Selbsteinschätzungen der Schüler*innen zur Bewältigung des Distanzunterrichts. Dies ist jedenfalls als Limitation zu nennen, auch wenn positive Befunde zur Reliabilität und Validität der Selbsteinschätzung von Schüler*innen vorliegen (Ross 2006). Wo möglich, wäre jedenfalls eine Triangulation derartiger Selbstauskunftsdaten wünschenswert. Aufgrund der regionalen Definition der Zielgruppe der Studie ist zudem die Frage der Generalisierbarkeit der Ergebnisse zu diskutieren. Methodisch basiert die Erhebung ausschließlich auf einem Fragebogen. Für diesen wurde, wo möglich, auf bestehende Inventare zurückgegriffen, welche für den konkreten Bedarf teils adaptiert wurden, teils wurden eigene Konstrukte erstellt. Damit gehen naturgemäß testtheoretische Grenzen einher. Da zudem im Fragebogen aufgrund des Studienkontexts ein breites Themenfeld abgedeckt werden musste, ist die Genauigkeit, mit der die im Beitrag diskutierten Konstrukte gemessen werden können, begrenzt. Gleichzeitig steht damit eine thematische Basis zur Verfügung, welche eine breitere Kontextualisierung der Befunde ermöglicht. Limitierend wirkt die im Fragebogen begrenzte Erfassung der konkreten Gestaltung des Distanzunterrichts und dessen Qualität. Der Übergang in die Sekundarstufe II ist – ganz unabhängig von den Herausforderungen der Pandemie – durch eine Vielfalt an Bedingungen moduliert, die in ihrer Gesamtheit nicht erfasst werden kann. Dementsprechend kann auch die vorliegende Studie nur einen Ausschnitt davon beleuchten.

## Fazit

Zusammenfassend ist festzuhalten, dass die diskutierten Regressionsanalysen für alle auf der Basis des Ressourcenmodells von Fend et al. (2009) berücksichtigten Ressourcenarten relevante Zusammenhänge mit der Bewältigung des Distanzunterrichts bestätigen. Basierend auf den Untersuchungsergebnissen kann der vorliegende Beitrag als Beleg für die Relevanz personaler Ressourcen hinsichtlich der Bewältigung des Distanzlernens interpretiert werden. Insbesondere den schulbezogenen personalen Ressourcen (dem schulischen Leistungsniveau sowie der Lernmotivation) kommt eine große Bedeutung zu. Schüler*innen, mit vergleichsweise geringen personalen Ressourcen schätzen ihre Bewältigung der Anforderungen des Distanzunterrichts im Schnitt weniger gut ein, was auf mangelnde Prozessgerechtigkeit (Fend 2019) hindeutet.

Zusammenhänge zwischen der Bewältigung des Distanzunterrichts können auch mit schulischen Faktoren gezeigt werden. Insgesamt ist die Erklärungskraft schulischer Ressourcen gegenüber den personalen jedoch gering. Grundsätzlich haben schulische Ressourcen das Potenzial, kompensatorisch in Bezug auf Unterschiede in den familiären Ressourcen sowie den individuellen Ausgangsbedingungen zu wirken. Hinsichtlich eines Gerechtigkeitsbegriffs, der auf Kompensation abzielt, wird ein Mehr an Förderung für Personengruppen, die unverschuldet Benachteiligungen erleben, als gerecht angesehen (Fend 2019). Der Schule kommt aus dieser Perspektive im Distanzunterricht insbesondere die Rolle zu, bestehende Benachteiligungen für die Bewältigung des Distanzlernens bestmöglich zu kompensieren. Vor diesem Hintergrund ist es als positiv zu werten, dass sich in den vorliegenden Analysen auch schulische Ressourcen als Einflussfaktoren auf die Bewältigung des Distanzunterrichts erweisen.

Die Befunde weisen darauf hin, dass Schulen die Entwicklung personaler Ressourcen stärker im Blick haben sollten. Strategien der Selbstregulation inkl. der Selbstorganisation und der Aufrechterhaltung von Motivation sind wesentliche Ressourcen für die Bewältigung von Lernanforderungen – grundsätzlich und im Besonderen bei leistungsschwachen Schüler*innen –, nicht nur im Setting des Distanzlernens. Zudem können Lehrpersonen nicht davon ausgehen, dass diese Bedingungen bei allen Schüler*innen in gleicher Form vorhanden sind. Für die Gestaltung von Distanzlernphasen ist demnach mit Blick auf leistungsschwächere und wenig motivierte Schüler*innen und solche mit niedrigen Lernkompetenzen eine gute Strukturierung und ggf. auch engmaschige Begleitung durch die Lehrpersonen zu planen und umzusetzen, um allen Schüler*innen adäquate Lerngelegenheiten zu bieten.

Schüler*innen mit niedrigem Leistungsniveau, niedriger Motivation und geringer Selbstwirksamkeitsüberzeugung stellen eine besonders vulnerable Gruppe im Distanzunterricht dar. Die eingangs angesprochene Vermutung von Bildungsforscher*innen, die Pandemie hätte eine verstärkende Wirkung auf Bildungsungerechtigkeit, wird von den im vorliegenden Beitrag diskutierten Befunden grundsätzlich gestützt. Es liegt die Schlussfolgerung einer Vergrößerung der Bildungsbenachteiligung durch die mit der Pandemie verbundenen Unterbrechungen des Präsenzunterrichts nahe. Insgesamt stellen breite Belege hierfür derzeit noch ein Desiderat dar.

## Footnotes

1 Bildungsgerechtigkeit bezieht sich laut Fend (2019) auf meritokratische Gerechtigkeit (ungleiche Belohnung ungleicher Merkmale [Leistung]), Prozessgerechtigkeit (gleiche Rechte auf Entfaltung von Talenten für alle) sowie kompensatorische Gerechtigkeit (ein Mehr an Förderung für unverschuldet Benachteiligte).
